# Semi-field experiments and sibship analyses reveal larval habitat size preferences and skip oviposition behaviour in female *Anopheles arabiensis*

**DOI:** 10.1186/s13071-026-07482-2

**Published:** 2026-06-09

**Authors:** Hudson Onen, Emmanuel W. Kaindoa, Perpetra Akite, Jonathan K. Kayondo, Martha A. Kaddumukasa, Anne M. Akol, Frederic Tripet

**Affiliations:** 1 Department of Zoology, Entomology and Fisheries Sciences, College of Natural Sciences, School of Biosciences, Makerere University, P.O. Box 7062, Kampala, Uganda; 2 Department of Entomology, Uganda Virus Research Institute, Plot 51/59 Nakiwogo Road, P.O. Box 49, Entebbe, Uganda; 3 Environmental Health and Ecological Science Department, Ifakara Health Institute, P.O. Box 53, Ifakara, Tanzania; 4 Faculty of Science, Biological Sciences, Kyambogo University, P.O. Box 1, Kampala, Uganda; 5 Centre for Applied Entomology and Parasitology, School of Life Sciences, Keele University, Staffordshire, UK; 6 Swiss Tropical and Public Health Institute, Kreuzstrasse 2, 4123 Allschwil, Switzerland; 7 Wits Research Institute for Malaria, School of Pathology, Faculty of Health Sciences, University of the Witwatersrand and the Centre for Emerging Zoonotic and Parasitic Diseases, National Institute for Communicable Diseases, Johannesburg, South Africa

**Keywords:** Oviposition, Site selection, Behaviour, *Anopheles arabiensis*, Habitats

## Abstract

**Background:**

Since the turn of the century, long-lasting insecticide-treated nets (LLINs) and indoor residual spraying (IRS) have played a major role in malaria control. However, the effectiveness of these tools is declining due to the development of insecticide resistance and other factors, creating an urgent need for complementary strategies. Larval source management (LSM), through the application of biological larvicides or the autodissemination of larvicides by ovipositing female mosquitoes, offers alternative avenues to target malaria vectors. Predicting the effectiveness of such approaches requires a detailed understanding of oviposition site selection behaviour in female *Anopheles gambiae* sensu lato. This study investigated the oviposition strategy of female *Anopheles arabiensis* in relation to aquatic habitat size under semi-field conditions in south-central Tanzania.

**Methods:**

An array of twelve alternating small (30 cm diameter, 20 L capacity) and large (60 cm diameter, 40 L capacity) artificial larval habitats was established in two compartments of a semi-field system. In quadruplicated experiments, forty wild gravid *An. arabiensis* females were released into each compartment and allowed to oviposit in their preferred habitats. The resulting third-instar larvae were collected, counted and preserved in ethanol. Larvae from one replicate were subjected to DNA extraction and microsatellite genotyping. Sibship analysis using Bayesian-likelihood methods (COLONY software) was conducted to reconstruct individual female oviposition behaviour.

**Results:**

Rather than distributing eggs in proportion to habitat size, *An. arabiensis* females significantly preferred smaller habitats. Sibship analysis showed that 49.2% of females skip-oviposited across two to four habitats, while others deposited eggs in only one. Skip-ovipositing females laid more eggs overall, but fewer eggs per habitat, and were more likely to alternate between small and large habitats than to use habitats of the same size, consistent with a potential bet-hedging strategy.

**Conclusions:**

Gravid *An. arabiensis* females exhibited a strong preference for smaller aquatic habitats and frequently engaged in skip-oviposition. These behavioural traits have potential implications for optimising larval-stage vector control tools, particularly those based on autodissemination and attract-and-kill strategies.

**Graphical Abstract:**

**Figure:**
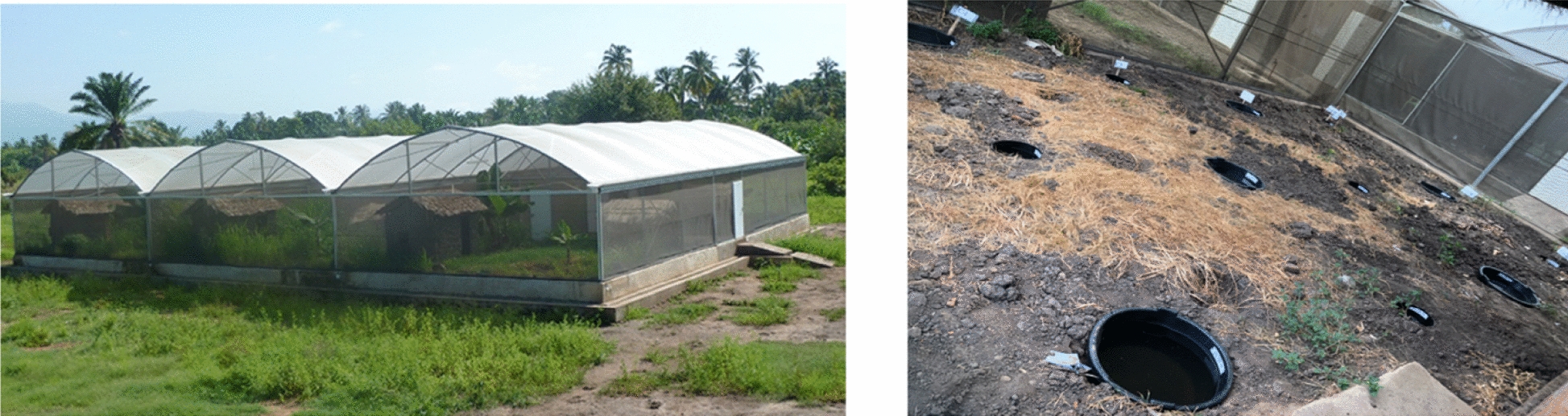

## Background

Enormous efforts have been made towards malaria elimination. Notably, an estimated 2.3 billion malaria cases and 14 million deaths were prevented in the African Region between 2000 and 2024 [1]. These successes were partly attributed to the widespread distribution of insecticide-treated bed nets (ITNs) and the application of indoor residual spraying (IRS) in areas of medium to high malaria transmission [1–3]. Despite these achievements, malaria reduction in many African countries has stalled owing to the declining effectiveness of ITNs and IRS [4]. The rapid spread of insecticide resistance and novel resistance mechanisms among major malaria vector species is undermining the impact of both the existing and newly developed chemical interventions [5, 6]. Additionally, declining LLIN coverage and reduced programme funding threaten the gains made in malaria control [7–9]. Consequently, there is a growing need for complementary vector control strategies that can sustain progress towards malaria elimination [10].

Larval source management (LSM), which targets mosquito larvae in aquatic habitats using microbial larvicides, such as *Bacillus thuringiensis israelensis* (Bti) and *Lysinibacillus sphaericus*, is receiving renewed attention as a complementary vector control tool [11–14]. Larval source management has often been considered a context-dependent intervention because its effectiveness strongly depends on the predictability and accessibility of mosquito larval habitats, which vary across ecological settings. However, recent developments in drone technologies and community-based implementation may create opportunities to expand its use, by decreasing the costs associated with deploying large numbers of dedicated personnel [15–17]. The effectiveness of LSM ultimately depends on a sound understanding of vector community dynamics, where gravid females of the main vector species choose to lay their eggs and which habitats contribute most to larval production [11].

Limited ecological information on oviposition site selection behaviour of key malaria vector species constrains the development of several promising LSM approaches. These include new larvicides, such as toxic synthesised nanoparticles or bio-larvicides derived from forest *Streptomyces* spp. [18, 19], attract-and-kill strategies that combine oviposition attractants with larviciding to target the most productive habitats [20–22], and autodissemination approaches in which gravid females transfer insect growth regulators (IGRs) such as pyriproxyfen, diflubenzuron or novaluron to oviposition sites [23–25]. All these strategies rely on a better understanding of the oviposition site selection behaviour by gravid malaria vector females, including how they distribute their eggs among habitats and the extent to which they engage in skip-oviposition behaviour, which remains poorly understood [26].

The major vectors of the *Anopheles gambiae* complex – *Anopheles gambiae* s.s., *An. coluzzii* and *An. Arabiensis* – are typically associated with small sunlit aquatic habitats such as hoof prints, rain puddles and irrigation furrows [27, 28]. In an extensive survey in Kenya, early instar larvae of *An. gambiae* s.l. were found at densities ranging from 161/m^2^ in puddles to 4/m^2^ in swamps and 9–10/m^2^ in cultivated swamps and open drainages [29, 30]. Temporary puddles are often shared by *An. gambiae* s.s., *An. arabiensis* and *An. coluzzii* [31], and may be favoured because they harbour fewer macroinvertebrate predators than larger, more permanent habitats [30].

Despite these general ecological patterns, relatively little is known about the behavioural strategy that gravid malaria vector females use when selecting oviposition sites. In pitcher plant mosquitoes, gravid females use habitat size as a proxy for habitat permanence and future resource availability, and thus prefer larger habitats that contain more larval food and are less prone to desiccation [32]. If gravid *An. arabiensis* females follow a similar adaptive habitat-matching strategy [33], larger habitats among other similar temporary pools might be expected to receive more eggs. However, oviposition decisions may also be influenced by other factors. A female mosquito typically lay between 20 and 290 eggs [34, 35], which may be distributed across multiple aquatic habitats through skip-oviposition behaviour [26].

The abundance of early larval instars in aquatic habitats is often used as a proxy for oviposition site preference by female mosquitoes and is usually assumed to reflect the contribution of multiple females selecting the same site [26, 36]. However, molecular ecological studies suggest that this assumption may be misleading. In western Kenya, genotyping of larvae at several microsatellite loci showed that *An. arabiensis* and *An. gambiae* s.s. females frequently share oviposition sites and that individual females often deposit only a few eggs per habitat [36, 37]. Similarly, approximately 50% of the gravid *An. arabiensis* females have been reported to skip-oviposit across multiple nearby artificial habitats or natural larval habitats [26]. Nevertheless, the behavioural mechanisms underlying the variation in larval abundance in relation to habitat size and skip-oviposition remain poorly understood.

In this study, we applied a genotype-based family reconstruction approach to investigate the oviposition behaviour of gravid *An. arabiensis* females in relation to two contrasting aquatic habitat sizes in a large semi-field system. Using microsatellite-based sibship analyses, we reconstructed the distribution of eggs laid by individual females across habitats, allowing us to quantify patterns of habitat use and skip-oviposition. On the basis of optimal oviposition theory, we hypothesised that gravid females exhibit adaptive habitat matching to maximise offspring survival. Under this framework, females might allocate eggs in proportion to habitat size, preferentially use smaller habitats owing to reduced predation risk and/or distribute eggs across multiple habitats through skip-oviposition. Understanding these behavioural strategies can improve predictions of larval habitat productivity and inform the design of larval source management strategies, including attract-and-kill and IGR autodissemination approaches.

## Methods

### Study area

**Fig. 1:**
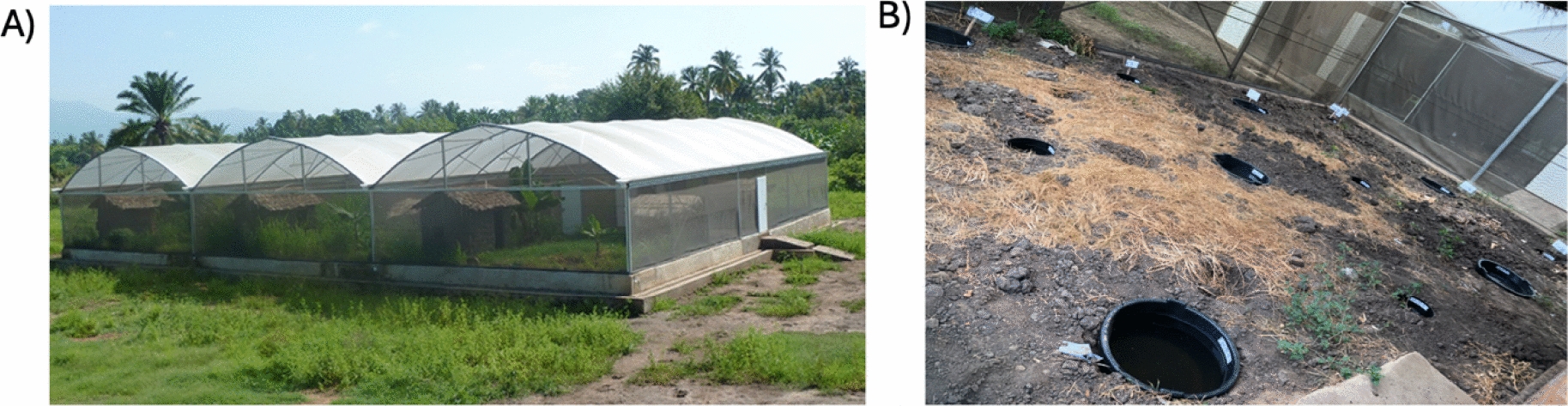
**a** Semi field enclosure; **b** randomized large and small artificial aquatic habitats in compartment 1 and 2

This study was conducted between November 2021 and March 2022 at the Ifakara Health Institute’s semi-field system (SFS) (Fig. 1a), located in Kining’ina village (8° 07′ 18″ S, 36° 39′ 06″ E) in south-eastern Tanzania. A detailed description of the SFS structure is available elsewhere [38]. For this experiment, two compartments of the SFS, each measuring 8.6 × 9 m, were used.

### Mosquito collections

Wild female *An. arabiensis* were collected inside houses using mouth aspirators and from outside using CDC light traps. Collections took place in the malaria-prone villages of Lupiro (8.41°S, 36.81°E) and Tulizamoyo (8.35447°S, 36.70546°E), where *An. arabiensis* is the sole species of the *An. gambiae* complex, following the local disappearance of *An. gambiae* s.s. over the past two decades [23]. Female mosquitoes were identified to genus level using morphological characteristics. Consequently, only *Anopheles* were transferred into mosquito field cages (15 × 15 × 15 cm), provided with 10% sugar solution soaked into a cotton pad placed on top of the netting enclosure. Mosquitoes were transported to the Ifakara Health Institute insectary at Kining’ina, starved for 12 h, blood-fed on chicken by placing its dressed ventral section on mosquito cage netting. Subsequently, the fully engorged mosquitoes were held for 72 h to allow for egg maturation.

### Preparation of artificial aquatic habitats

In each of the two SFS compartments (Fig. 1a), six small (30-cm diameter, 20-L capacity) and six large (60-cm diameter, 40-L capacity) artificial aquatic habitats were prepared by embedding black basins into the soil at 2 m intervals, arranged in three rows and four columns following a fully randomised design. Forty-eight hours before the start of the experiment, the 24 artificial aquatic habitats were filled with well water and 5 g of sieved adjacent soil was added, then left to condition for 48 h under semi-field conditions (Fig. 1b). Aquatic predators were removed from the SFS and were never observed in the artificial aquatic habitats.

### Gravid female larval habitat size preference experiments

#### Mosquito release in the semi-field enclosure

Two sets of 40 visibly gravid female mosquitoes were aspirated from the field collection cages using mouth aspirators. Each set was transferred into its respective paper cup through a hole punched through non-insecticide-treated net secured with a rubber band. Mosquitoes were provided with a 10% sugar solution and held at room temperature for 2 h. Each set was released at the centre of its designated semi-field compartment (Fig. 1a) by gently opening the lid of the paper cup, allowing mosquitoes to fly out freely.

#### Larval collection and preservation

At 3 days after mosquito release, habitats in both compartments were checked for first-instar larvae. Habitats in which 1 larva or more were observed were considered “used” while those with no larvae as ‘unused’. To facilitate downstream processing, larvae were left in the habitats for an additional 48 h until they moulted to the third instar (L3). This stage was targeted because the larger body size simplifies handling and provides sufficient DNA for genetic analyses. During this period, habitats were fed daily with 0.8 g of Tetramin Baby fish flakes to minimise resource competition among larvae. On day 5, all L3 larvae from each habitat were collected using a Pasteur pipette and counted to estimate larval abundance. Larvae were then killed in 100% ethanol for 2 min and transferred to labelled 2 ml screw-cap cryovials containing 80% ethanol. Samples were subsequently shipped to the Centre for Applied Entomology and Parasitology at Keele University (UK) for molecular species identification and microsatellite analysis.

## Data analyses

Oviposition site preference experiments were performed in quadruplicate. Data from the full factorial design were analysed using JMP version 14 software (SAS Institute, Inc., USA). The effect of habitat size, replicate and compartment on the proportion of habitats used by females was analysed using nominal logistic regression with a binomial distribution. Inferred egg counts from L3 larvae numbers were analysed using generalised linear models with a Poisson distribution and overdispersion. For all analyses, data were tested for continuity, normality and variance homogeneity, and analysed using parametric or nonparametric methods accordingly.

### Sib-families reconstruction via molecular analyses

#### Larval DNA extractions

Genomic DNA was extracted using the DNAzol protocol [39] with minor modifications. Briefly, individual larvae were placed in 1.5 ml microcentrifuge tubes containing 100 µL of DNAzol solution (Life Technologies Ltd, UK), then homogenised with a pestle until no body parts were visible. The homogenate was centrifuged at 10,000 *g* for 10 min, and the supernatant was transferred to a new tube. DNA was precipitated with 50 µL of 100% ethanol, gently shaken 5–8 times, incubated at room temperature for 3 min and centrifuged at 7000 *g* for 7 min. After discarding the supernatant, the pellet was washed with 750 µL of 75% ethanol. Excess ethanol was removed after a brief centrifugation, and 100 µL of TE buffer (10 mM Tris, 1 mM ethylenediaminetetraacetic acid [EDTA], pH 8.0) was added. DNA quantity and purity were assessed using a Nanodrop 2000 spectrophotometer (Thermo Scientific, UK). The extracted DNA was used for species identification and microsatellite genotyping for sibship reconstruction.

#### Molecular species identification

Species identification was performed using the SINE200 polymerase chain reaction (PCR) diagnostic assay, which distinguishes *An. coluzzii*, *An. gambiae* s.s. and *An. arabiensis* [40, 41]. Amplification was conducted using a PCRmax Alpha Thermal Cycler (Cole-Parmer Ltd, UK). The reaction mixture contained 0.5 µL of each forward (5′-TCGCCTTAGACCTTGCGTTA-3′) and reverse (5′-CGCTTCAAGAATTCGAGATAC-3′) primer, 10 µL of 2× Red Taq master mix, 8 µL of nuclease-free water and 1 µL of DNA template, resulting to a final volume of 20 µL. Thermocycling conditions were: 94 °C for 10 min; 35 cycles of 94 °C for 30 s, 54 °C for 30 s, 72 °C for 1 min; and a final extension at 72 °C for 10 min. PCR products were visualised on 1.5% agarose gel using Syngene system (Synoptics Ltd, UK).

#### Microsatellite genotyping

Microsatellite genotyping and sibship analyses were conducted on larvae collected from compartments 1 and 2 of the first replicate. Nine microsatellite loci were used: AG3H119, AG3H59, AG3H812, AG3H249 and AG3H555 (on chromosome 3R); AG3H127, AG3H242, AG3H577 and AG3H817 (on chromosome 3L) [42, 43]. These loci were selected to avoid known chromosomal inversions and regions of reproductive isolation on the X chromosome [44, 45]. PCR amplification conditions were: 94 °C for 10 min; 35 cycles of 95 °C for 30 s, locus-specific annealing temperature for 30 s (Table 1), 72 °C for 2 min; and a final extension at 72 °C for 10 min. PCR products labelled with different fluorescent dyes were grouped into three triplexes on the basis of predicted allele size and compatibility, to avoid peak overlap, and sent for genotyping at Eurofins Scientifics (Ebersberg, Germany).

**Table 1:** Sequence and dye used in microsatellite loci triplexes optimized for genotyping and sibship analyses – the expected allele size [43] and best annealing temperature

Triplex	Locus	Primer sequence and dye labels^a^	Allele size	Anneal. temp
1	AG3H249	[ATTO565] F: ATGTTCCGCACTTCCGACAC R: GCGAGCTACAACAATGGAGC	129	64.5 °C
	AG3H555	[FAM] F: GCAGAGACACTTTCCGAAAC R: TGTCAACCCACATTTTGCGC	81	60.2 °C
	AG3H817	[YAKYE] F: ACTGGTCCGTTGCTGCGCG R: ATGAGTGAATGGTGCGCTGG	124	65.1 °C
2	AG3H127	[YAKYE] F: CCTCTAACTCGATTACCGTG R: GTCAGGGAATTGGAAAGAGC	84	51.6 °C
	AG3H242	[FAM] F: TTCATTTCCACCGCAGCTGC R: GGCGACACTCAATCCTTCC	69	53.8 °C
	AG3H577	[ATTO565] F: TTCAGCTTCAGGTTGGTCTC R: GGGTTTTTTGGCTGCGACTG	113	61.6 °C
3	AG3H59	[YAKYE] F: CCCCTATTAAACCCTGGACG R: TGTTGTTGCCCTGCGTTACC	123	55.2 °C
	AG3H119	[FAM] F: GGTTGATGCTGAAGAGTGGG R: ATGCCAGCGGATACGATTCG	174	60.7 °C
	AG3H812	[ATTO565] F: CTGGCCCATTTTGCATATGC R: TGCTCCACCCAAACCACATC	131	59.4 °C

^a^[ATTO565] corresponds to red, [FAM] to blue and [YAKYE] to green, fluorescent labels on forward primers

#### Microsatellite allele size calling and genetic diversity

Allele sizes from each locus were manually scored and allocated to the same allelic size bins used in previous population genetic studies that used these loci, i.e., AG3H249 [46], AG3H555, AG3H817, AG3H127, AG3H242, AG3H577, AG3H59, AG3H119 and AG3H812 [43]. Allele richness and genotypic diversity were computed using JMP software version 14 (SAS Institute, Inc., USA). The expected and observed numbers of homozygotes and heterozygotes were calculated using Levene’s correction from the online version of the GENEPOP software (https://genepop.curtin.edu.au/genepop_op5.html) and used to calculate heterozygosity for each locus.

#### Maximum likelihood sibship analyses

On the basis of the multilocus genotypes, sibship among mosquito L3 larvae across habitats was determined using the Bayesian Maximum Likelihood software COLONY (https://www.zsl.org/about-zsl/resources/software/colony) [47], enabling reconstruction of the number of gravid females that laid eggs and their individual oviposition decisions. Family inferences were calculated under the assumption of a monogamous mating system, a medium run length and a default seed number of 1234. Preliminary analyses considered none, weak, medium and strong prior strengths, with male and female prior values of 0, 10, 20 and 30, respectively, as well as different microsatellite typing error rates. Thereafter, sibling/family membership was inferred using weak prior values for a 20:20 male-to-female ratio with a genotyping error rate of 0.05, which were the parameter values that generated the best likelihood estimates. The COLONY output assigns each larvae to a full-sib family inferred from the multilocus genotype data. Each inferred sibship was assumed to correspond to the progeny of a single gravid female because females of the *An. gambiae* complex typically mate once. Accordingly, each reconstructed family was treated as a unique mother ID. For each mother ID, the number of larvae recovered in each habitat was quantified, allowing reconstruction of the spatial distribution of eggs laid by individual females across habitats within each compartment. These reconstructed oviposition patterns were then used to determine whether females deposited eggs in a single habitat (non-skip oviposition) or distributed eggs among multiple habitats (skip oviposition). The data from compartments 1 and 2 were analysed independently.

## Results

### Habitat size effect

**Fig. 2:**
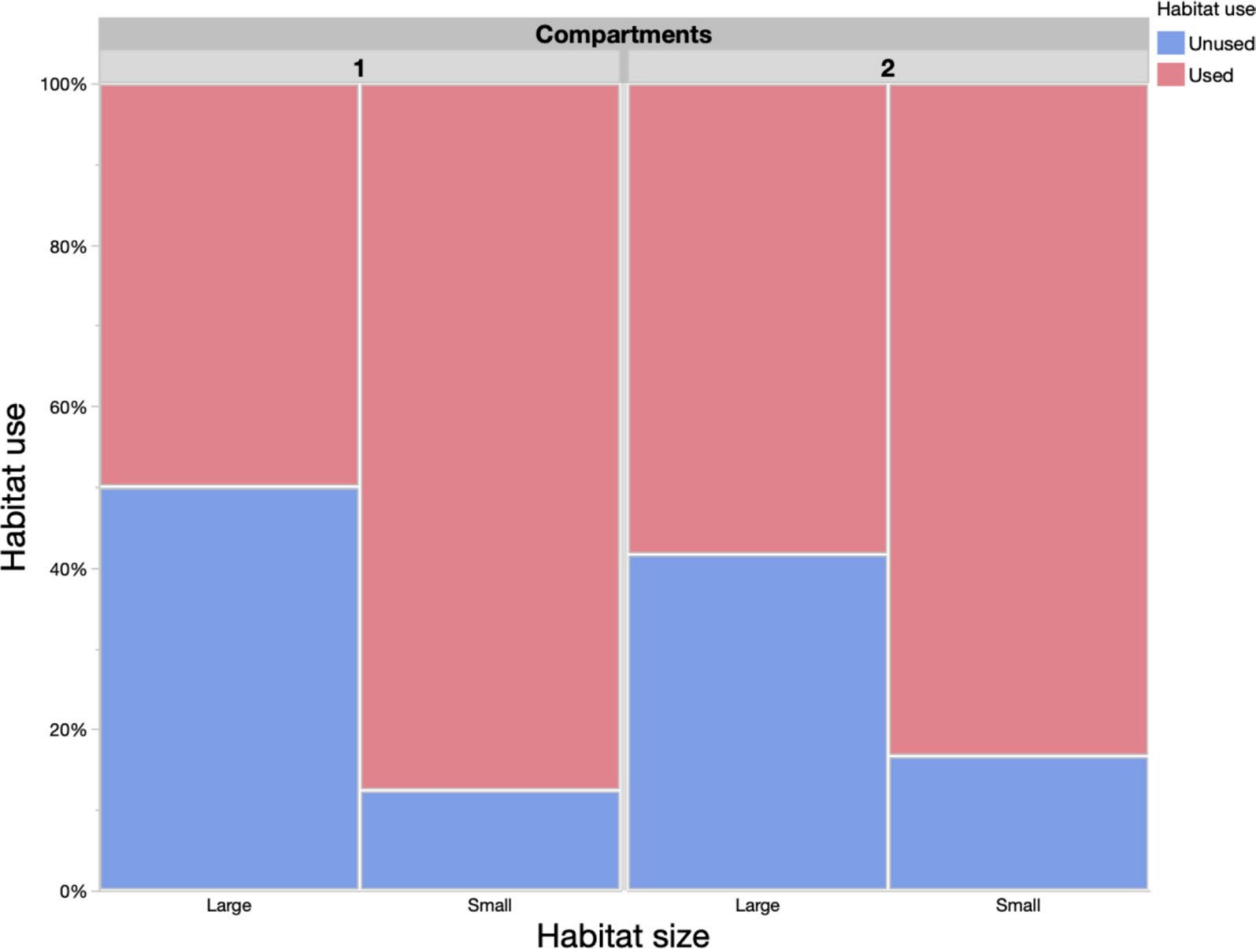
Effect of habitat size on artificial habitat use by gravid *An. arabiensis* females

As expected, all the mosquito larvae obtained were confirmed molecularly as *An. arabiensis*. Habitat size significantly influenced the proportion of habitat used by gravid *An. arabiensis* females (logistic regression model: L-R chi-square = 11.54, *n* = 96, *P* = 0.007), and there was no effect of compartments (L-R chi-square = 0.056, *n* = 96, *P* = 0.813). Overall, 85.4% of small habitats were used compared with 54.2% of larger-sized habitats (Fig. 2; Table 2), and a total of 2280 larvae were recovered from small habitats compared with 726 from larger habitats.

**Table 2:** Percentage habitats used, mean and total number of L3 larvae across habitat size, replicates and compartments

Habitat size/replicate	Compartment 1				Compartment 2				Both compartments			
	N	% used (95% CIs)	Mean L3 (95% CIs)	Total L3	N	% used (95% CIs)	Mean L3 (95% CIs)	Total L3	N	% used (95% CIs)	Mean L3 (95% CIs)	Total L3
Replicate 1												
Large	6	66.7 (29.9–90.3)	27.2 (0.3–54.1)	163	6	66.7 (29.9–90.3)	33.2 (−2.9 to 69.2)	199	12	66.7 (39.1–86.2)	30.2 (11.7–48.6)	362
Small	6	83.3 (43.6–96.9)	32.3 (10.9–53.8)	194	6	83.3 (43.6–96.9)	36.7 (−0.2 to 73.4)	220	12	83.3 (55.2–95.3)	34.5 (17.1–51.9)	414
All treatments	12	75.0 (66.8–91.1)	29.8 (15.6–43.9)	357	12	75.0 (66.8–91.1)	34.9 (13.9–55.94)	419	24	75.0 (55.1–88.0)	32.3 (20.6–44.0)	776
Replicate 2												
Large	6	33.3 (9.7–70.0)	2.7 (−3.2 to 8.6)	16	6	33.3 (9.7–70.0)	5.3 (−3.9 to 14.6)	32	12	33.3 (13.8–60.9)	4.0 (−0.6 to 8.6)	48
Small	6	100 (60.9–100)	51.8 (26.4–77.3)	311	6	66.7 (29.9–90.3)	45.2 (5.6–84.8)	271	12	83.3 (55.2–95.3)	48.5 (29.2–67.8)	582
All treatments	12	66.7 (39.1–86.2)	27.3 (7.8–46.7)	327	12	50.0 (25.4–74.6)	25.3 (4.0–46.5)	303	24	58.3 (38.8–75.5)	26.3 (12.9–39.5)	630
Replicate 3												
Large	6	50.0 (18.8–81.2)	9.8 (−6.8 to 26.5)	59	6	50.0 (18.8–81.2)	18 (−3.5 to 39.5)	108	12	50.0 (25.4–74.6)	13.9 (2.5–25.3)	167
Small	6	83.3 (43.6–96.9)	38.7 (8.0–69.3)	232	6	100 (60.9–100)	60.3 (17.3–103.3)	362	12	91.7 (64.6–98.5)	49.5 (26.8–72.2)	594
All treatments	12	66.7 (39.1–86.2)	24.3 (7.1–41.4)	291	12	75.0 (66.8–91.1)	39.2 (15.0–63.3)	470	24	70.8 (50.8–85.1)	31.7 (17.7–45.7)	761
Replicate 4												
Large	6	50.0 (18.8–81.2)	10.2 (−11.1 to 31.4)	61	6	83.3 (43.6–96.9)	14.7 (5.9–23.4)	88	12	66.7 (39.1–86.2)	12.4 (2.9–21.9)	149
Small	6	83.3 (43.6–96.9)	57.3 (21.7–92.9)	344	6	83.3 (43.6–96.9)	57.7 (18.0–97.3)	346	12	83.3 (55.2–95.3)	57.5 (35.7–79.3)	690
All treatments	12	66.7 (39.1–86.2)	33.8 (10.7–56.8)	405	12	83.3 (55.2–95.3)	36.2 (14.3–58.0)	434	24	75.0 (55.1–88.0)	34.9 (20.3–49.6)	839
All replicates	48	68.8 (54.7–80.0)	28.8 (20.4–37.1)	1380	48	70.8 (56.8–81.8)	33.9 (23.9–43.8)	1626	96	69.8 (60.1–78.1)	31.3 (24.9–37.7)	3006

**Fig. 3:**
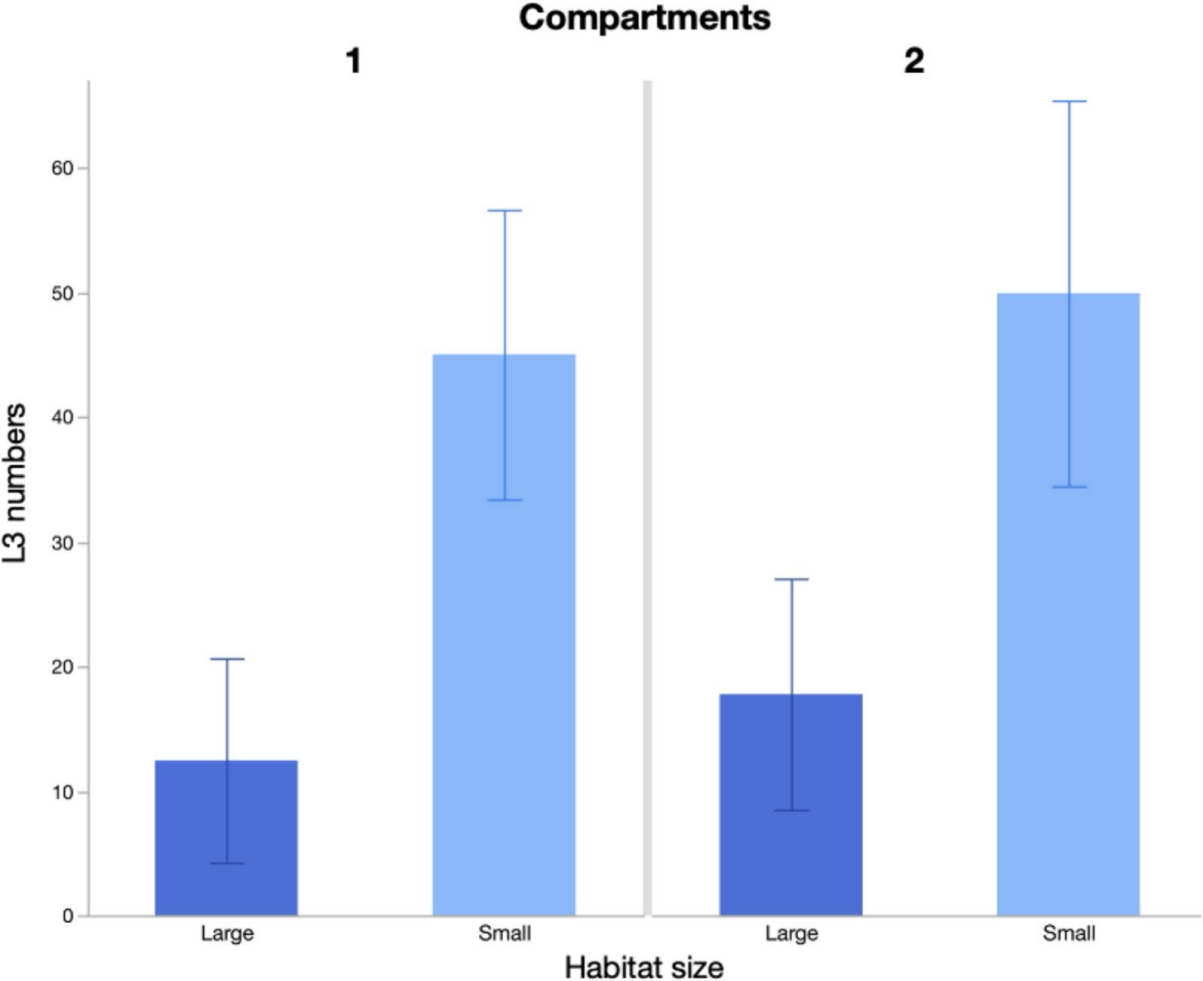
Effect of habitat size on the abundance of *An. arabiensis* L3 in both SFS compartments

Small habitats also contained significantly higher number of L3 larvae per habitat than larger ones (General Linear Model: L-R chi-square = 33.84, *n* = 96, *P* < 0.001). There was no significant effect of compartment on the number of larvae recovered (L-R chi-square: = 0.808, *n* = 96, *P* = 0.372). Overall, the minimum number of eggs laid per habitat across both compartments inferred from L3 larval numbers averaged to 31.3 (95% confidence interval [CI] 24.9–37.7) (Table 2) and ranged from 0 to 102. In small habitats the mean number was 47.50 (95% CI: 38.15–56.85) compared with 15.13 (95% CI 9.11–21.14) in larger ones (Fig. 3).

### Maximum likelihood sibship analyses

#### Microsatellite allele richness, diversities and heterozygosity

Microsatellite genotyping towards sibship analyses was performed on all L3 larvae from the first replicate of the experiment (Table 3). The maximum allelic richness and genotypic diversity were recorded from loci AG3H812 and AG3H249 and the minimum were from loci AG3H127. However, the maximum heterozygosity was recorded from locus AG3H119, and minimum was with locus AG3H127 (Table 3).

**Table 3:** Allelic richness, diversity and heterozygosity of the microsatellite loci used

Microsatellite loci	Chromosome	Position (bp)	Allelic richness	Genotypic diversity	Heterozygosity
AG3H59	3R	4,239,709	13	30	0.76
AG3H812	3R	6,204,540	16	36	0.74
AG3H249	3R	8,791,931	16	37	0.57
AG3H119	3R	14,828,212	11	16	0.67
AG3H127	3L	16,858,957	5	6	0.14
AG3H577	3L	19,767,127	12	32	0.79
AG3H555	3R	21,302,535	9	24	0.56
AG3H817	3L	31,854,973	9	12	0.44
AG3H242	3L	37,130,620	9	16	0.32

### Sib-families reconstruction

**Fig. 4:**
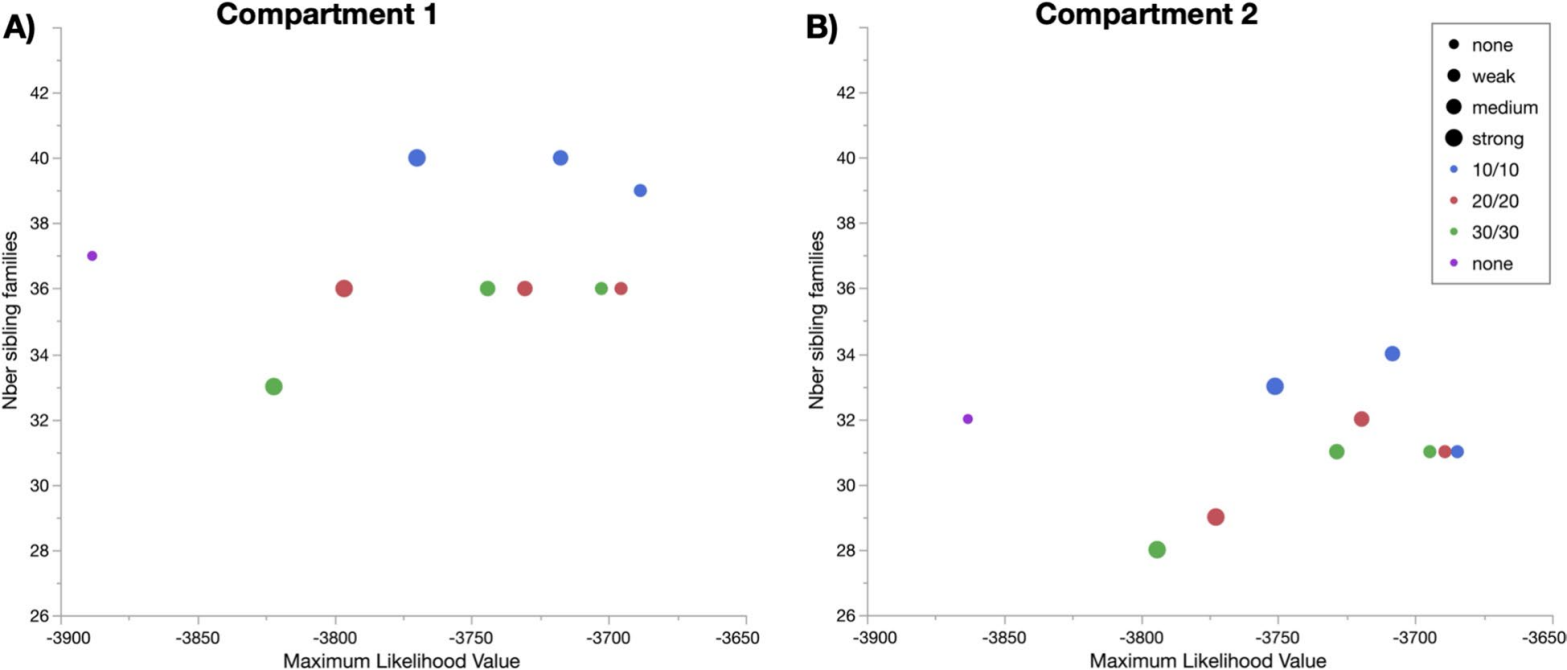
Number of sibling families inferred from L3 larvae genotypes by maximum likelihood in relation to prior values (male-to-female ratio) and prior strengths used for: **A** compartment 1 and **B** compartment 2

For both SFS compartments, inferring the number of females/families from all L3 larval genotypes using COLONY software was most consistently performed with a weak prior value for a 20:20 male-to-female ratio, which resulted in the highest maximum likelihood values (Fig. 4A, B). Thirty-six families were inferred in compartment 1 and 31 families in compartment 2, suggesting that 90% and 77.5% of the 40 gravid females laid eggs on the night following their release (Fig. 4A, B).

### Inferred female skip-oviposition behaviour

**Fig. 5:**
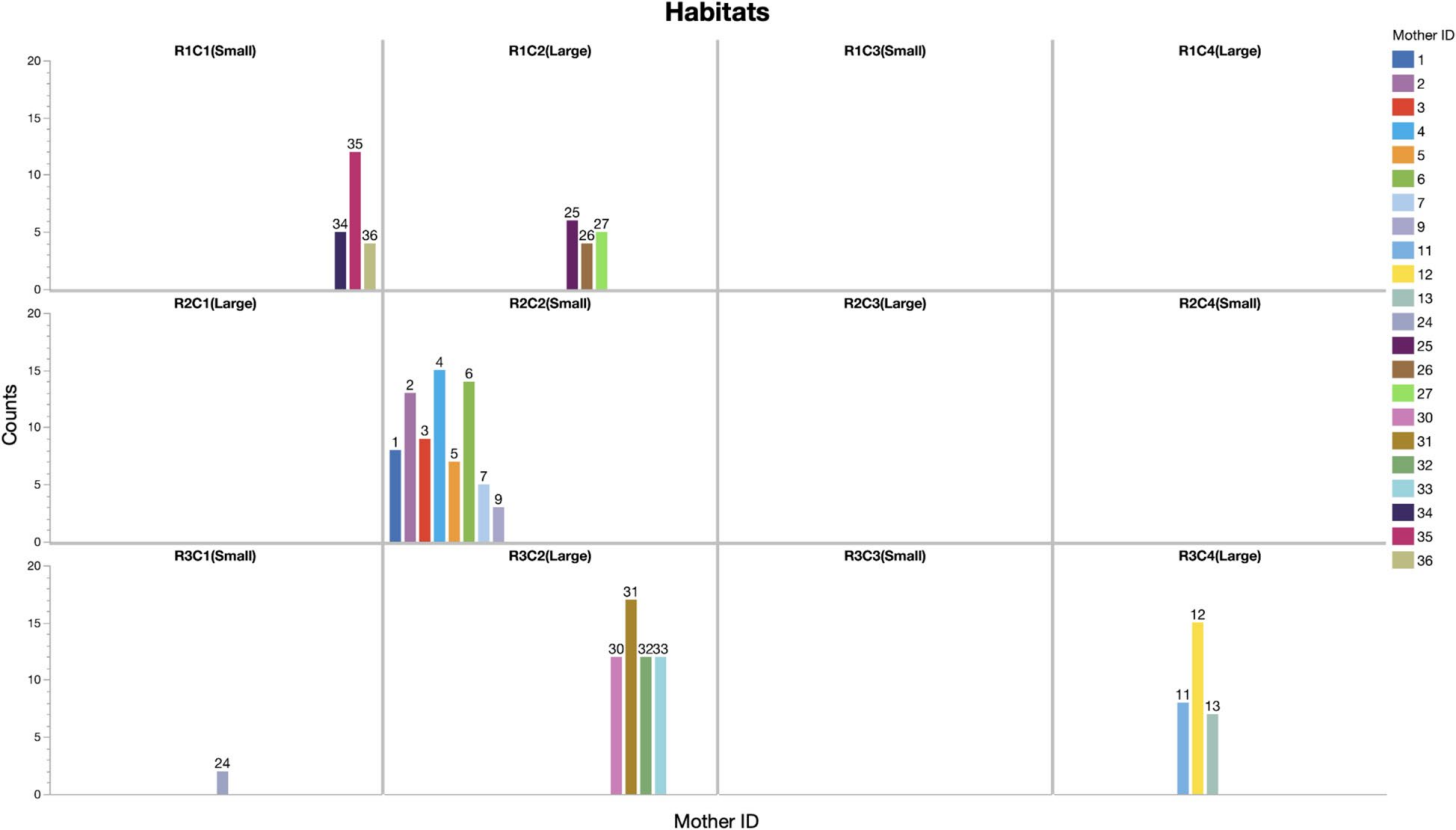
Inferred distribution of eggs laid by 22 non-skip-ovipositing *An. arabiensis* females among small and large habitats in compartment 1

**Fig. 6:**
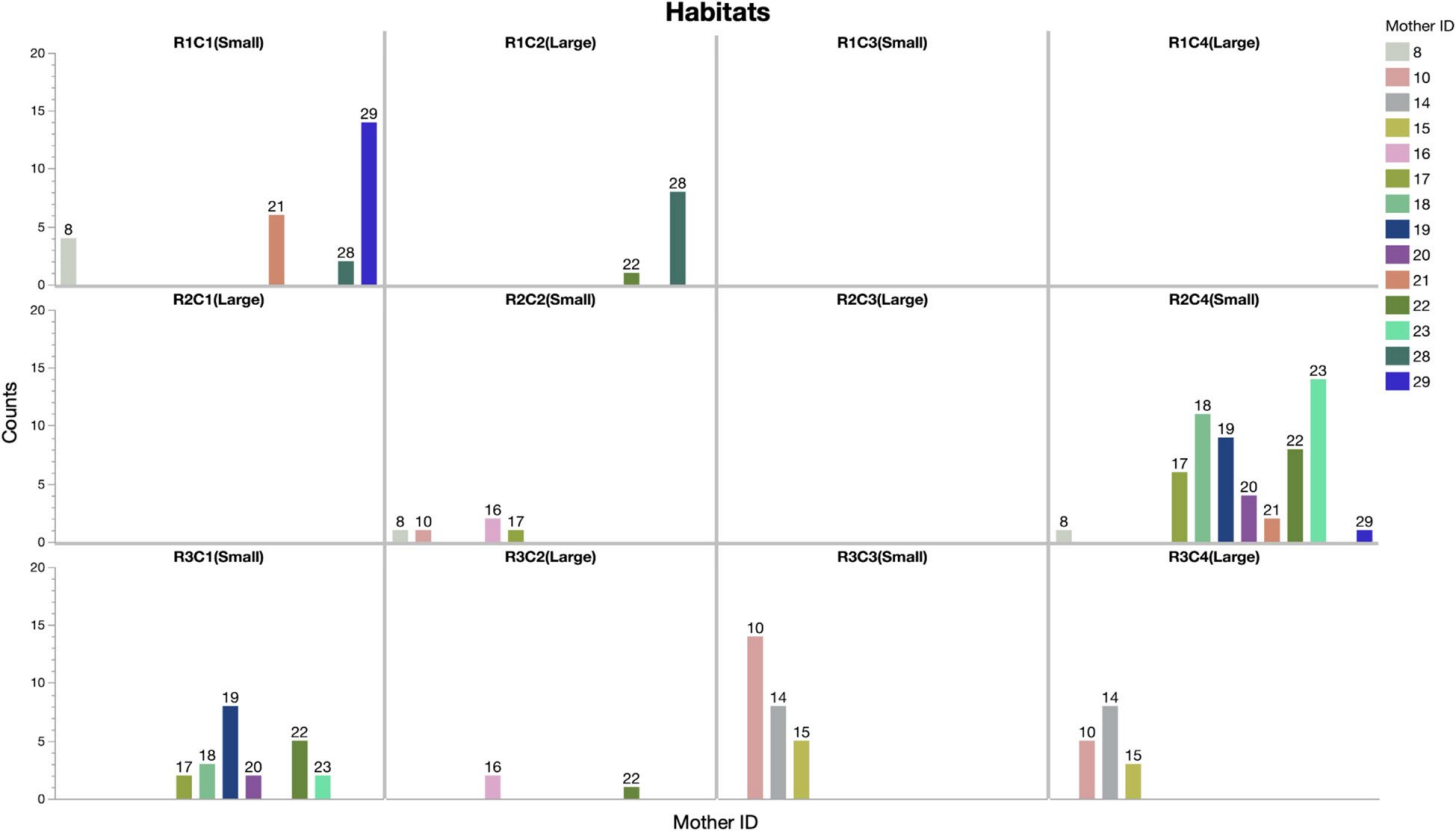
Inferred distribution of eggs laid by 12 non-skip ovipositing *An. arabiensis* females among small and large habitats in compartment 2

**Fig. 7:**
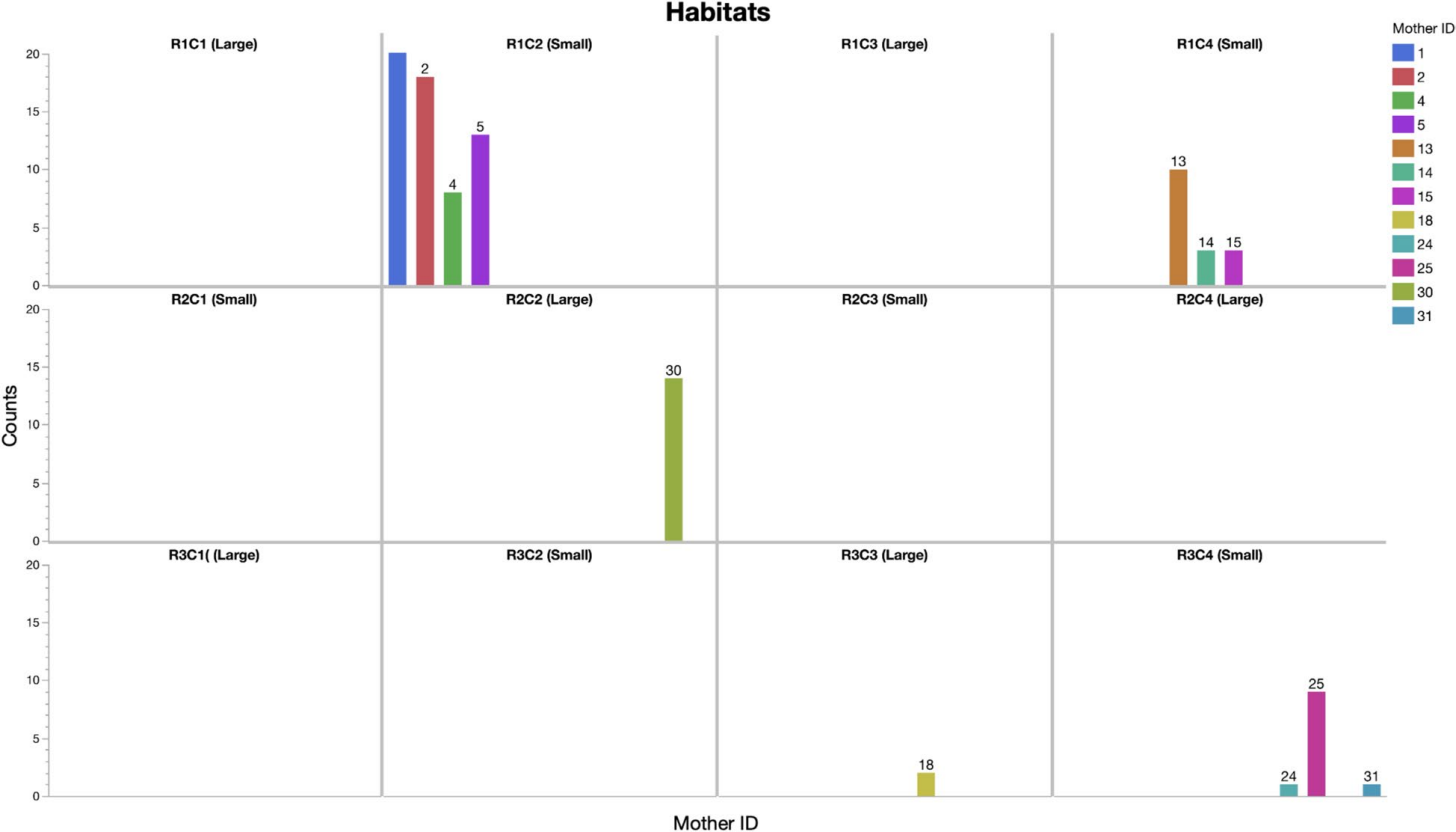
Inferred distribution of eggs laid by 14 skip-ovipositing gravid *An. arabiensis* across habitat in compartment 1 – mother identification codes found in more than 1 habitat indicates oviposition in those habitats

**Fig. 8:**
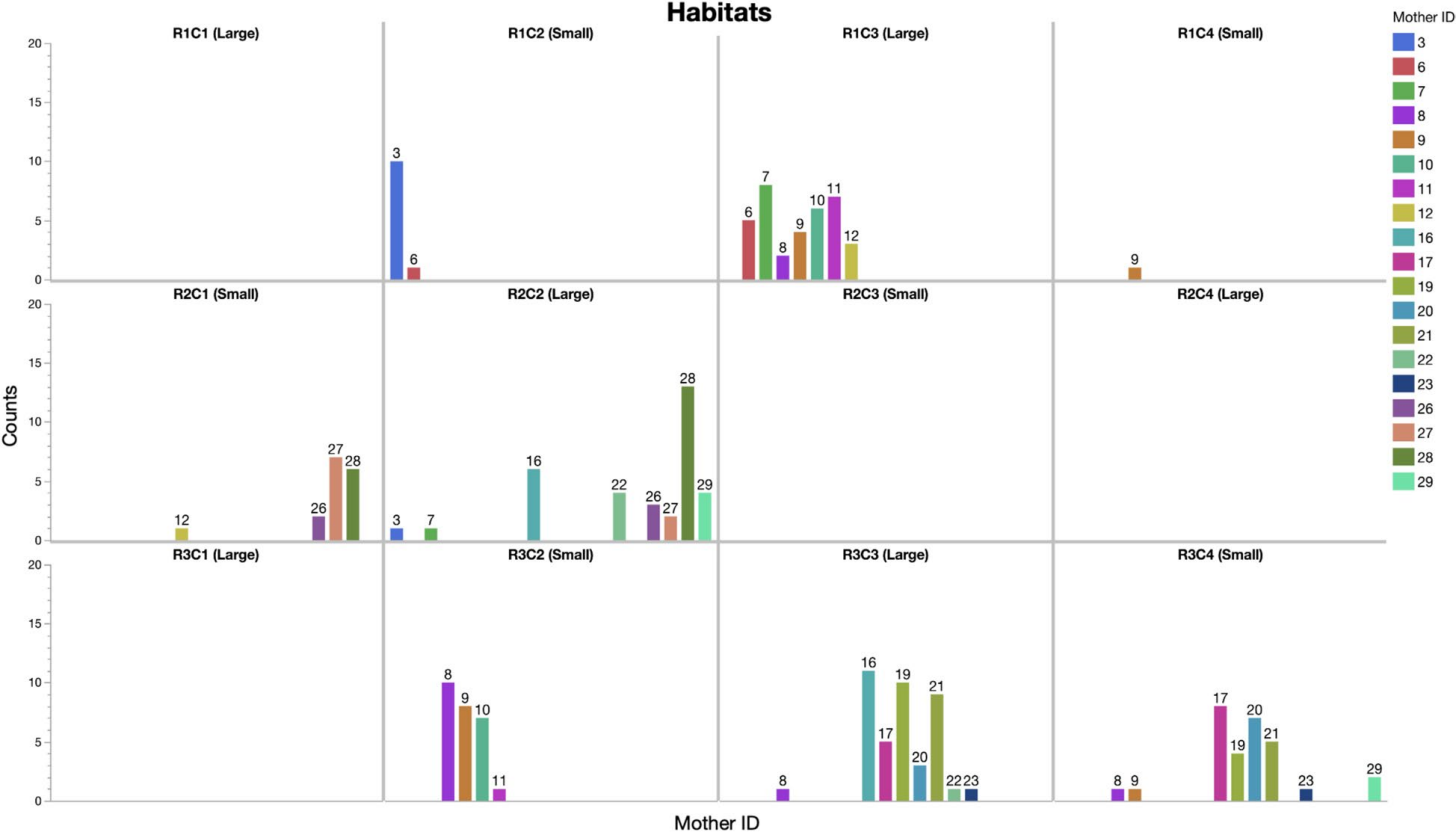
Inferred distribution of eggs laid by 19 skip-ovipositing gravid *An. arabiensis* across habitat in compartment 2 – mother identification codes found in more than 1 habitat indicates oviposition in those habitats

**Fig. 9:**
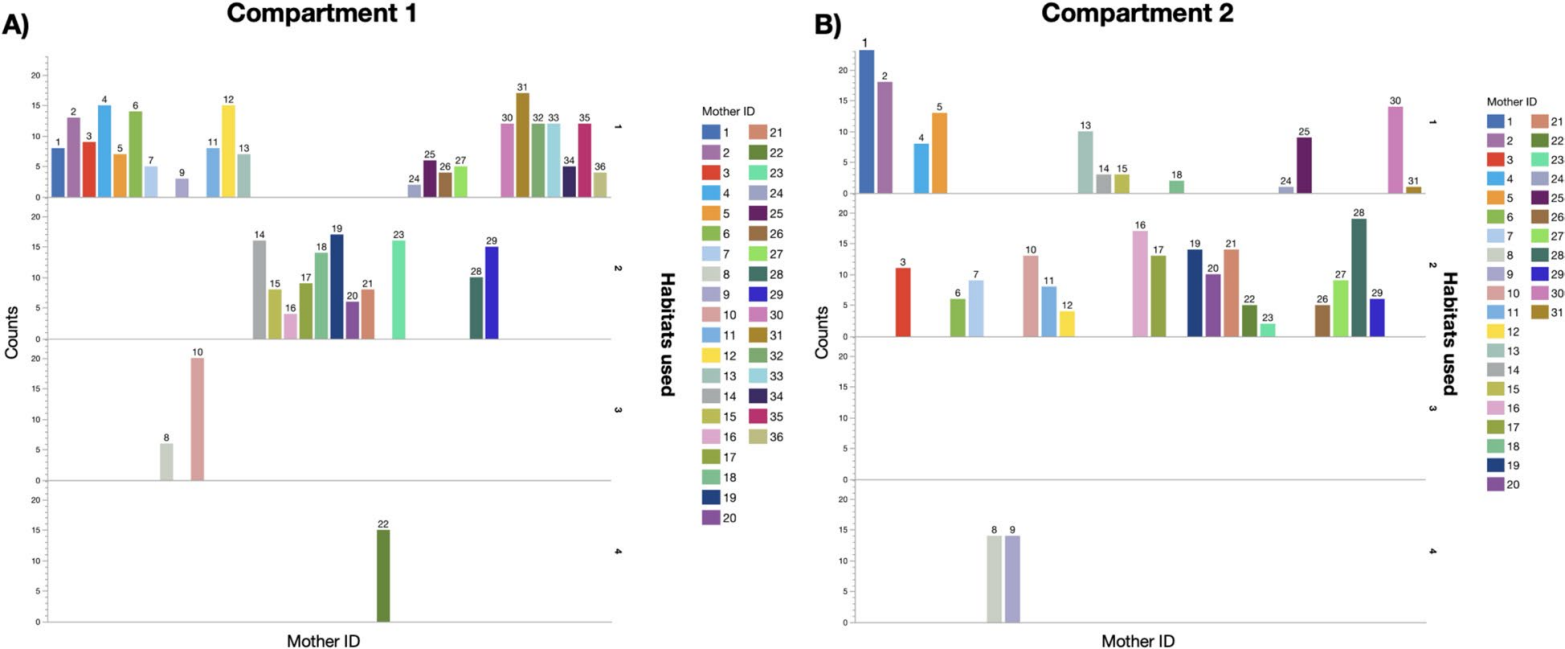
Overall habitat uses by gravid *An. arabiensis* in **A** compartment 1 and **B** compartment 2

In both compartments, sibship analyses revealed that some gravid females, herein referred to ‘non-skip ovipositor’, laid eggs in a single habitat (Figs. 5, 6), while others, referred to as ‘skip-ovipositors’, laid eggs in more than one habitat overnight (Figs. 7, 8). In compartment 1, 14 out of 36 (38.9%) skip oviposited. In compartment 2, this proportion was higher with 19 out of 31 (61.9%) skip-ovipositors albeit non-significant (chi-square Likelihood ratio: *χ*^2^ = 3.37, *df* = 1, *P* = 0.066). Some skip-ovipositing females laid eggs in more than two habitats. In compartment 1, 11 females skip-oviposited in two habitats, 2 in three habitats and one female laid egg in four habitats (Fig. 9A). In compartment 2, 12 gravid females did not skip oviposit, 17 skip oviposited in two habitats and two skip-oviposited in 4 habitats (Fig. 9B).

### Skip-oviposition and number of eggs laid

Overall, females laid a median of 9 (interquartile range [IQR] 5–14) eggs (range 1–35) and there was no significant differences between compartments in terms of eggs laid (Wilcoxon two-sample test: *Z* = 0.40, *n* = 67, *P* = 0.691). Skip-ovipositing females laid a median of 10 (6–14.5) eggs, which was significantly higher than non-skip-ovipositing females 8 (4–13) (Wilcoxon two-sample test one-sided: *Z* = 1.76, *P* = 0.039). However, skip-ovipositing females laid significantly fewer eggs per habitat than non-skip-ovipositing females with a median of 4 (1–8) compared with 8 (4–13) (Wilcoxon two-sample test: *Z* = 3.83, *n* = 109, *p* < 0.001) (Table 4).

**Table 4:** Percentage habitats used, median and total number of eggs laid by skip and non-skip-ovipositing females in different sized habitats and two SFS compartments inferred from L3 numbers

Behaviour/habitat size	Compartment 1			Compartment 2			Both compartments		
	*N*	Median eggs (IQR)	Total eggs	*N*	Median eggs (IQR)	Total eggs	*N*	Median eggs (IQR)	Total eggs
Non-skip females									
Large	10	10 (5.8–12.8)	98	2	8 (2–14)	16	12	10 (5.3–13.5)	114
Small	12	7.5 (4.3–12.8)	97	10	8.5 (2.5–14.3)	101	22	8 (3–13)	198
All habitats	22	8 (5–12.3)	195	12	8.5 (2.3–13.8)	117	34	8 (4–13)	312
Skip females									
Large	7	3 (1–8)	28	23	4 (2–7)	110	30	4 (1.8–7.3)	138
Small	26	4 (2–8)	136	19	4 (1–7)	83	45	4 (1–8)	219
All habitats	33	4 (2–8)	164	42	4 (1–7)	193	75	4 (1–8)	357
All females									
Large	17	7 (3.5–12)	126	25	4 (2–7.5)	126	42	5 (2–8.3)	252
Small	38	5 (2–9)	233	29	5 (1–8.5)	184	67	5 (2–9)	417
All habitats	55	5 (2–9)	359	54	4 (1–8)	310	109	5 (2–8.5)	669

### Effect of habitat size on oviposition behaviour

Overall, a larger proportion of small habitats were used than larger ones (Likelihood ratio chi-square: *χ*^2^ = 5.79, *P* = 0.016). However, among used habitats, there was no significant difference in the number of females having oviposited in small and larger habitats as inferred from L3 larvae sibship analysis (Wilcoxon two-sample t test; *Z* = −0.384, *n* = 16, *P* = 0.701).

Habitat size did not influence the number of eggs laid overnight into them by individual gravid females (Wilcoxon two-sample test: *Z* = 0.198, *n* = 109, *P* = 0.840). This was also true when only considering skip-ovipositing females (Wilcoxon two-sample *t* test; *Z* = −0.016, *n* = 75, *P* = 0.987) (Table 4). The proportion of skip-ovipositing gravid females that first laid eggs in a small habitat and then in a large habitat (or the reverse) was 68.0%, thus higher than laying twice in small habitats (24.0%), or twice in larger habitats (8.0%) (L-R chi-square: *χ*^2^ = 14.59, *n* = 25, *P* < 0.001), demonstrating that skip-ovipositing females may ‘bet hedge’ by ovipositing in two habitat sizes, and overall prefer smaller habitats.

### Skip oviposition distance between habitats

Using the data from the 26 skip-ovipositing females that laid in two habitats, we calculated the proportion that consecutively laid eggs in closest adjacent habitats as opposed to exploring and laying habitats further away. Fourteen females laid eggs in adjacent habitats (positioned to the left, right, in front or behind), as opposed to 11 laying eggs further away (at a diagonal or further away), thus there was no significant preference for shorter distances (L-R chi-square: *χ*^2^ = 0.36, *n* = 26, *P* = 0.548). Females laying in three or four habitats always laid in combinations of habitats that involved some habitats further than directly adjacent.

## Discussion

This study provides insight into how gravid *An. arabiensis* females optimise oviposition strategy across habitats of contrasting sizes under semi-field conditions in south-eastern Tanzania. Females strongly preferred smaller-sized habitats compared with larger ones set in the two semi-field compartments. This resulted in a much higher proportion of the smaller habitats being used, and many more L3 larvae being recovered from them compared with the larger ones. Detailed sibship analyses using nine polymorphic microsatellite markers and the Bayesian maximum likelihood software COLONY confirmed the preference for smaller habitats at the individual female level. They further revealed that females laid few eggs per habitat regardless of habitat size but laid a higher total number of eggs when skip-ovipositing. In addition to preferring smaller habitats, individual skip-ovipositing females appeared to oviposit in manner consistent with a potential bet-hedging strategy by laying eggs in both habitat sizes.

The fact that *An. arabiensis* females preferred smaller habitats is consistent with several surveys of natural larval habitats, which have shown that *An. gambiae* s.l. favours smaller habitats [48–51]. A recent comprehensive survey of larval habitats in a rice-growing region of Côte d’Ivoire reported that over two-third of the identified aquatic habitats were <1 m^2^, including many smaller pools within rice paddies [52]. In this study, the artificial habitats were small compared with some of those in natural settings, with surface areas of 0.071 m^2^ for the smallest and 0.283 m^2^ for the larger ones. These sizes nevertheless fall within the range of many naturally occurring *An. gambiae* s.l. habitats, such as hoof prints, rain puddles and rock pools. When offered the choice, gravid females strongly preferred smaller habitats. Such preference may be explained by the fact that larvae in smaller, temporary pools are exposed to fewer predators than those in larger, more permanent habitats [30, 53]. Consequently, laying in the smaller suitable habitats may be the best strategy. This choice is further supported by the species’ skip-oviposition behaviour [54].

Skip oviposition has been detailed by three molecular ecological studies using microsatellite markers, demonstrating that *An. gambiae* s.l. females tend to lay few eggs and that many spread their eggs across several habitats [26, 36]. In this and previous studies, when presented with multiple nearby habitats, approximately 50% of females skip-oviposited [26, 36]. That skip-oviposition was associated with females laying more eggs but fewer per habitat, that females skip-oviposited preferentially in adjacent habitats and that they were more likely to skip between small and larger habitats, or use two small habitats, all point to a complex adaptive oviposition behaviour.

The female’s optimal behaviour would depend on her mature egg load and the availability and size of larval habitats, while also minimising flight distances. Only a few skip-ovipositing females used three or four habitats, distributing eggs over a much larger section of the 8.6 × 9 m semi-field compartment. While the compartment dimensions were ideally suited to reveal oviposition site choice behaviour, it remains unknown whether females first search the area for oviposition sites before initiating egg laying or sequentially decide to lay a certain number of eggs in the first suitable aquatic habitat encountered. This may depend on the distance at which females become aware of oviposition sites and the resolution at which they can achieve this. Gravid females are thought to rely primarily on detecting water vapour and associated semiochemicals to identify oviposition habitats several metres away [22, 55]. Once near a site, females may sample the water with their tarsi and proboscis, using gustatory receptors to assess quality, as recently demonstrated in *Ae. Albopictus* [56]. This would allow them to detect additional cues, such as the presence of predators or competitors [57–59], availability of additional food such as pollen in the habitat [60, 61].

This study had several limitations. Firstly, experiments were conducted in a semi-field system that intentionally reduced ecological complexity to isolate the effect of habitat size on oviposition behaviour. In natural settings, additional factors, such as predators and environmental variability, may interact with habitat size preference and further influence oviposition behaviour. Secondly, siblingship analyses were conducted on a single replicate of the experiment which may not capture variability among cohorts. Third, for genetic analyses, larvae were allowed to grow to the L3 stage to ensure sufficient DNA could be extracted for microsatellite analysis. Even under optimal conditions (with food supplied and no predators), some larval mortality is expected [62]. Additionally, egg hatch rate can vary. Thus, in general, using larval counts as proxy for egg numbers generally leads to underestimation of female fecundity. Here, females were also blood-fed on chicken blood, which is known to reduce fecundity and longevity compared with human blood [63, 64]. Therefore, some of our inferences about egg laying are more qualitative than quantitative and could have resulted in an underestimation of skip-oviposition. Analytically, the software COLONY has been reported to overestimate the number of *An. gambiae* sib-families compared with other approaches [26]. In our study, estimates of family numbers were unrealistically high until we increased the genotyping error rate to 5%, which resulted in convergence of sibship numbers and higher likelihoods across different prior conditions.

The widespread occurrence of skip-oviposition and preference for small habitats may have important implications for LSM. When resources are limited, smaller habitats could be prioritised. Attract-and-kill strategies may particularly benefit from this, as targeting smaller habitats requires less attractant and toxicant while potentially achieving disproportionately high impact. In this context, skip-oviposition could greatly enhance the effectiveness of autodissemination approaches. Furthermore, the fact that females readily use small artificial habitats suggests that, in some settings, the strategic deployment of treated artificial habitats may offer a viable alternative to the labour-intensive search and treatment of natural habitats – enabling dissemination of larvicides via skip-ovipositing females.

## Conclusions

Oviposition behaviour has direct consequences for mosquito fitness. Female mosquitoes optimise egg distribution to enhance offspring survival [54, 65]. Here, females did not distribute eggs in proportion to habitat size, deviating from a simple habitat matching rule [33]. Instead, they laid a few eggs per habitat, and many skip-oviposited. Females with larger egg loads were more likely to skip-oviposit, and skip-ovipositing females appeared to distribute their eggs in ways consistent with bet-hedging by laying in habitats of contrasting sizes. This complex oviposition strategy highlights suggests behavioural flexibility of the trait while offering valuable insights for LSM, particularly pull-and-kill and autodissemination approaches.

## Data Availability

The datasets analysed during the study are available from the corresponding author upon reasonable request.

## Abbreviations

LSM: Larval source management
L3: Third larval instars
PCR: Polymerase chain reactions
SFS: Semi field system
CDC: Centre for Disease Control
ITN: Insecticide treated nets
IRS: Indoor residual spraying
LLIN: Lonag lasting insecticide treated nets
DNA: Deoxyribonucleic acid
